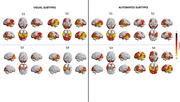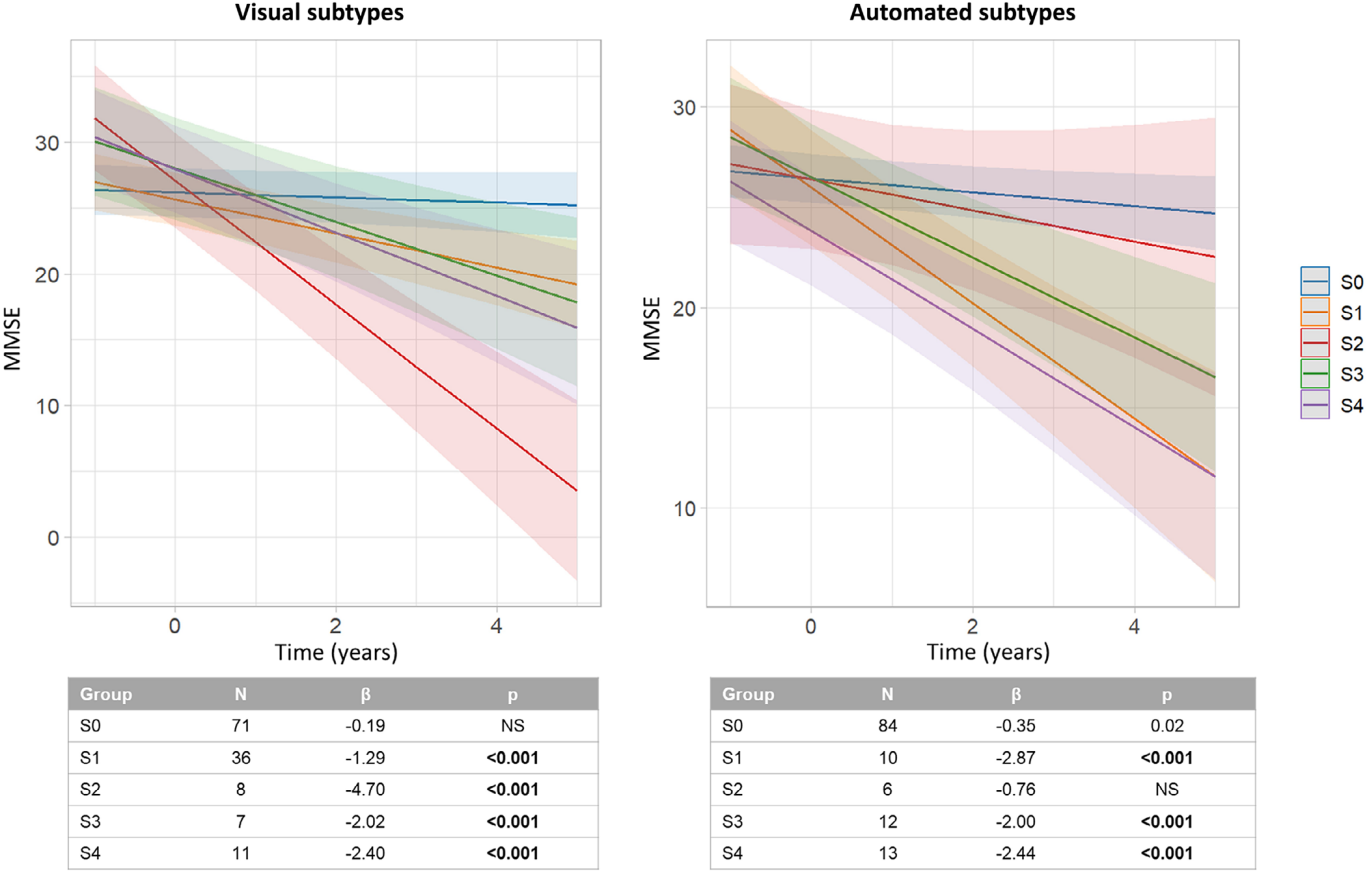# Visual classification of tau‐PET detects four subtypes with different long‐term outcomes

**DOI:** 10.1002/alz.087373

**Published:** 2025-01-09

**Authors:** Cecilia Boccalini, Gregory Mathoux, Ines Hristovska, Federica Ribaldi, Débora Elisa Peretti, Annachiara Arnone, Max Scheffler, Giovanni B. Frisoni, Oskar Hansson, Jacob W. Vogel, Valentina Garibotto

**Affiliations:** ^1^ University of Geneva, Geneva Switzerland; ^2^ Geneva University Hospitals, Geneva Switzerland; ^3^ Clinical Memory Research Unit, Department of Clinical Sciences, Lund University, Lund Sweden; ^4^ Geneva Memory Center, Department of Rehabilitation and Geriatrics, Geneva University Hospitals, Geneva Switzerland; ^5^ Laboratory of Neuroimaging and Innovative Molecular Tracers (NIMTlab), University of Geneva, Neurocentre and Faculty of Medicine, Geneva Switzerland; ^6^ Laboratory of Neuroimaging and Innovative Molecular Tracers (NIMTlab), Geneva University Neurocenter and Faculty of Medicine, University of Geneva, Geneva, Switzerland (2) Memory Clinic, Geneva University Hospitals, Geneva, Switzerland (3) Nuclear M, Geneva Switzerland; ^7^ Division of Radiology, Geneva University Hospitals, Geneva Switzerland; ^8^ Memory Clinic, Geneva University Hospitals, Geneva Switzerland; ^9^ Department of Clinical Sciences Malmö, SciLifeLab, Lund University, Lund Sweden; ^10^ Division of Nuclear Medicine, Geneva University Hospitals, Geneva Switzerland

## Abstract

**Background:**

Substantial variability in tau accumulation patterns in Alzheimer’s disease (AD) population has now become accepted. Subtype and Stage Inference (SuStaIn) has distinguished four distinct spatiotemporal trajectories of tau pathology: limbic (S1), medial temporal lobe‐sparing (S2), posterior (S3), and lateral temporal (S4). A visual method to validate and identify them is a requirement for their clinical translation. Our study aims to provide evidence for multiple tau accumulation patterns in a clinical setting by developing and testing a novel topographic visual method for tau‐PET based on SuStaIn subtypes.

**Method:**

We included 245 participants from the Geneva Memory Clinic, who underwent ^18^F‐Flortaucipir‐PET. All scans were classified into different subtypes by visual ratings and the automated SuStain algorithm. Cohen's kappa (k) tested the agreement between raters and between visual and automated subtypes. Chi‐squared and Kruskal‐Wallis tests were used to test differences in clinical features, tau, and amyloid (Aβ) loads between subtypes. Differences in cognitive trajectories were tested using linear mixed‐effects models, controlling for age, sex, and tau stages.

**Result:**

A substantial agreement between raters was found in visually interpreting tau pattern subtypes (k>0.65, p<0.001) and a fair agreement between visual and automated subtypes (k=0.39, p<0.001), with the automated approach detecting a higher rate of negative scans. According to the visual classification, individuals with S2 subtype were younger than S1 and S3 (p<0.001), had worse MMSE and verbal fluency scores (p<0.05) than S4 and S1, showed higher global tau than other subtypes (p<0.05), and a steeper cognitive decline.

**Conclusion:**

Our results show that visual classification can reliably identify four tau patterns, differing in global tau loads, clinical characteristics, and long‐term outcomes, suggesting its clinical usefulness for the detection of higher‐risk AD variants. A clinically implementable classification in subtypes with faster decline is paramount for personalized diagnosis and therapy.